# Insights in wound healing properties of water-soluble composition of dihydroquercetin and L-lysine

**DOI:** 10.3389/jpps.2025.13831

**Published:** 2025-03-12

**Authors:** Artem A. Svotin, Amir Taldaev, Ilya D. Nikitin, Maria D. Korochkina, Roman P. Terekhov, Irina A. Selivanova

**Affiliations:** ^1^ Nelyubin Institute of Pharmacy, Sechenov First Moscow State Medical University, Moscow, Russia; ^2^ Laboratory for the Study of Single Biomacromolecules, Institute of Biomedical Chemistry, Moscow, Russia; ^3^ Research Center for Molecular Mechanisms of Aging and Age-Related Diseases, Moscow Institute of Physics and Technology, Dolgoprudny, Russia; ^4^ A. A. Kharkevich Institute for Information Transmission Problems Russian Academy of Sciences, Moscow, Russia

**Keywords:** dihydroquercetin, flavonoid, L-lysine, burn, wistar rats

## Abstract

This study focuses on implementing a composition of the natural flavonoid dihydroquercetin (DHQ) with L-lysine in the treatment of thermal burns. The wound-healing activity of DHQ is well-known. The addition of amino acid to the composition increases the water solubility of the flavonoids, providing an opportunity to develop a spray dosage form. The research involved 60 male Wistar rats divided into five treatment groups. Sea buck oil served as a positive control. On day 14, the composition treatment group showed significant progress in wound healing, being 9.6 ± 2.0% ahead of the other groups in absolute terms. On day 35, treatment with the composition resulted in a significant decrease in relative wound area to 1.9 ± 0.9%, while in the negative and positive control groups, it was 10.7 ± 7.8% and 8.4 ± 4.9%, respectively. At the same time, the epidermal and dermal layers were found to be clearly distinguished in the composition treatment according to histological analysis. Numerous collagen fibres were clearly visible, and the active process of keloid scar formation was observed. An additive effect of the combined use of DHQ and L-lysine was observed (*F* = 0.21, *p* = 0.649). A natural next step is to develop the dosage form for the DHQ-L-lysine composition.

## Introduction

Despite a general downward trend in the number of burns in developed countries [1], this type of injury remains a problem for the healthcare system. According to the WHO, 180,000 heat-related deaths occur each year, with the majority taking place in low- and middle-income countries [2]. In 2019, nearly 9 million cases of burns of varying severity were registered worldwide [3]. Furthermore, such injuries are often characterised by a small area of damage and mild pathological processes that do not require qualified medical care. Therefore, it is impossible to assess the true epidemiology of this condition.

At present, scientists are mostly focusing on treating severe forms of burns using cellular and transplantation methods. Recently, much attention has been paid to the possibility of stem cell therapy [4–6]. However, such technologies are expensive, which limits their use in outpatient medical practice.

**FIGURE 1:**
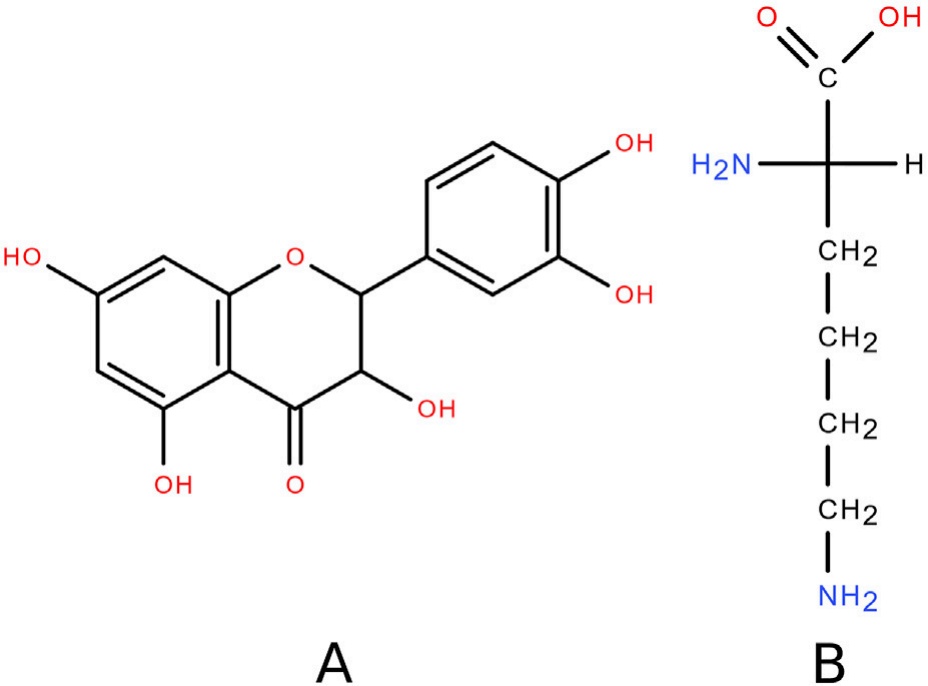
Structural formula of **(A)** DHQ and **(B)** lysine.

Natural compounds that can be used in the treatment of skin lesions deserve special attention. One example of such compounds is dihydroquercetin (DHQ) – 2,3-dihydro-3,5,7-trihydroxy-2-(3,4-dihydroxyphenyl)-4*H*-1-benzopyran-4-one – the structure of which is shown in Figure 1A. This substance is a major flavonoid component of the wood of Siberian larch (*Larix sibirica* Ledeb.) and Dahurian larch (*Larix dahurica* Turcz.) [7]. Skin regenerative activity [8, 9], and anti-inflammatory effects [10–12], have been previously demonstrated for DHQ.

This flavonoid is characterised by poor water solubility [13] at ambient temperature and is classified as “very slightly soluble” according to the State Pharmacopoeia of the Russian Federation XV, which is equivalent to the terms of the Pharmacopoeia of the Eurasian Economic Union [14] and the European Pharmacopoeia 11.5 [15] due to harmonisation. This makes it difficult to develop new DHQ-based remedies. However, this substance has been shown to have a high safety profile [16–18].

Increasing the solubility of biologically active compounds is a challenge in pharmaceutical science. Various approaches have been suggested to solve this problem. For example, using cocrystallisation [19, 20], a pseudo-polymorph was synthesised in the form of a hemihydrate [21]. New amorphous forms have been obtained using various drying technologies [22, 23]. In some cases, the water solubility reached the “soluble” category according to the Pharmacopoeia of the Eurasian Economic Union. Solid dispersions of DHQ with various polysaccharides have often been described [24–26].

The object of the present study is a water-soluble (“very easily soluble” according to the Pharmacopeia of the Eurasian Economic Union) composition of DHQ with the proteinogenic amino acid L-lysine – (*S*)-2,6-diaminohexanoic acid (Figure 1B). L-lysine is an essential amino acid found in natural products of animal [27, 28] and plant [29, 30] origin. The structure of this compound is characterised by two amino functional groups that can form hydrogen bonds with the acidic centres of DHQ, resulting in better water solubility of the flavonoid [31, 32]. In a systematic review, it was shown that the adverse events of L-lysine supplementation were not significant, and the safe dose for humans was found to be 6.0 g/d [33]. There is evidence [34] suggesting that L-lysine may be beneficial in anti-inflammatory therapy by reducing the levels of TNF-α, IL-8, and MIF. Taken together, these data suggest the potential benefits with low safety risks of adding L-lysine to an anti-burn formulation with DHQ.

Therefore, the aim of this study is to research the wound-healing activity of the DHQ-L-lysine composition.

## Methods

### Animals

The study was performed on 3-month-old male Wistar rats (*n* = 60; mean weight 270 g) provided by the vivarium of Sechenov University (Russia). The animals were kept in groups of six individuals in polycarbonate cages with metal-hinged lids at a temperature of 23 ± 2°C and a relative humidity of 50%–60%. They had free access to water and food, which were replaced and replenished daily. The cage bedding consisted of wood shavings, which were changed every 2 days. These conditions comply with the requirements of the European Convention for the protection of vertebrate animals used for experimental and other scientific purposes (ETS N 123) [35].

### Experimental design

Seven days before the formation of the burn the animals were randomly distributed into 5 groups and then underwent acclimatisation for 7 days.

For randomisation, an online random number generator was used, which was given a sequence from 1 to 60. The number drawn was assigned to the animal, and the values themselves were not repeated. Rats with numbers 1–12 formed group I, 13–24 – group II, and so on.

Group I is a negative control group. No specific treatment was applied. The burn surfaces were moistened with 1 mL of distilled water every day.

Group II is a positive control group. Daily treatment was performed with 1 mL of sea buckthorn seed oil (Tula Pharmaceutical Factory LLC, Tula, Russia). This herbal medicine has been approved for the treatment of burns.

In group III the burns were treated with 1 mL of DHQ suspension (99.1%, Ametis JSC, Blagoveshchensk, Russia) at a concentration of 50 mg/mL once a day. This dose of DHQ has been assessed in a previous study [9] and has been shown to be effective in wound healing.

In group IV, a 1 mL solution of L-lysine monohydrate (Pharmaceutical grade, AppliChem GmbH, Darmstadt, Germany) at a concentration of 50 mg/mL was applied to the burn surface every day.

In group V, the treatment consisted of daily topical application of 1 mL of a composition solution, containing 50 mg/mL of DHQ. The ratio of flavanonol to L-lysine monohydrate was 50:50 by weight (approximately 1:2 in moles). To prepare the composition solution, the components were pulverised together in a porcelain mortar for 10 min. The mixture was then dissolved in the necessary amount of water.

**FIGURE 2:**
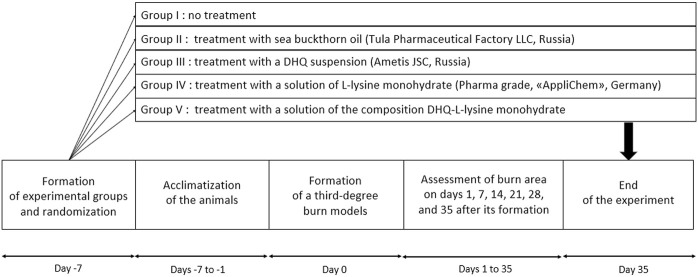
Experimental design.

On days 1, 7, 14, 21, 28, and 35 after the formation of the burn, body weights and temperatures were measured (Figure 2). Wound healing was controlled by the Popova contact method [36]: A transparent plastic film was placed over the burn, the edge of the wound was outlined using ink, and then its area was calculated using graph paper. The results of the measurements were presented in absolute and relative values. The last parameter was calculated as the percentage of the burn area to the initial one on day 1.

### Establishment of the rat third-degree burn model

To create a third-degree burn, a common technique was used [37–39]. Briefly, a 200 g metal plate with a contact area of 5 cm^2^ was heated to 105 ± 5°C. It was then placed for 20 s on the shaved back of a rat, which was deeply anaesthetised with the drug “Zoletil” (Virbac, Carros, France).

### Histological analysis

On days 7, 14, 21, 28, and 35, the wound surface was harvested at the border with healthy tissue under anaesthesia with ethyl ether (Kuzbassorgkhim LLC, Kemerovo, Russia). Haemostasis was achieved with 3% hydrogen peroxide (Samaramedprom JSC, Chapaevsk, Russia). The histological sample was fixed in a 10% buffered formalin solution (BioVitrum, Saint-Petersburg, Russia) and then embedded in paraffin. Sections of 2 μm thickness were cut using a Leica RM 2265 microtome (Nussloch, Germany) and then placed in a warm water bath to remove paraffin. They were then transferred to glass slides and placed on a Leica HI 1220 heating table (Nussloch, Germany). The obtained samples were stained with haematoxylin and eosin according to the manufacturer’s standard protocol (BioVitrum, Saint-Petersburg, Russia) using a Leica Autostainer XL staining machine (Nussloch, Germany). Stained samples were placed in a Leica SUB-X mounting medium (Nussloch, Germany) for mounting under a coverslip on an automated Leica CV5030 device (Germany). Microslides were analysed using a Leica DM2000 microscope (Leica Microsystems, Wetzlar, Germany) equipped with a ToupCam UCMOS03100KPA digital camera (Hangzhou, China) at ×400 magnification.

Representative micrographs were used for qualitative histological analysis.

### Ethical statement

The design of this experiment was approved by the Ethics Committee of Sechenov University (protocol No. 19-20, July 02, 2020).

### Semi-quantitative analysis

Semi-quantitative analysis was performed by scoring histochemically stained tissues and cells. To assess the intensity of the labelled stains, the approximate number of visible plasma cells, fibroblasts, and collagen fibres was calculated. The counting was performed by one observer. These data were then analysed independently, without relying on the results of the histological analysis. The types of inflammatory processes and their phases on different days were therefore classified [40].

### Statistics

The data are presented as mean ± half-width of the confidence interval and statistically analysed by one-way analysis of variance (ANOVA) with significant difference at *p* < 0.05 using Tukey’s multiple comparison *post hoc* test. To clarify the significance of the results, a comparison between the study groups was made simultaneously.

To assess the reduction in the burn surface area, absolute values and relative values (RV) were used:

$$RV=\frac{\sum_{i=1}^{n}\frac{S_{i_{n}}}{S_{i_{1}}}}{n}\times 100\%,$$

where $S_{i_{n}}$ is the absolute burn surface area for *i* individual rat in group I (or II, III etc) on day *n*, $S_{i_{1}}$ is the absolute burn surface area for *i* individual rat in group on day 1, and *n* is the number of animals in a group.

To establish the nature of the pharmacological interaction between DHQ and lysine, a two-way ANOVA test was performed in the Origin Pro 2018 software. Values of *p* < 0.05 were considered to be significant.

## Results

Measurements of body weight and temperature were used to assess the initial condition of the experimental animals.

The average weight of the experimental animals before the start of the experiment was 267.1 ± 5.4 g (*p* < 0.05). However, no significant difference was found between the formed groups (Table 1).

**TABLE 1:** Weight of experimental animals.

Group	Average weight ± half-width of the confidence interval, g						
	Day 0	Day 1	Day 7	Day 14	Day 21	Day 28	Day 35
I	272.3 ± 12.9	266.2 ± 12.3	261.4 ± 14.0	266.1 ± 14.1	268.4 ± 14.9	273.1 ± 16.8	272.8 ± 13.9
II	268.3 ± 14.7	263.1 ± 15.4	258.8 ± 15.0	275.5 ± 19.5	270.0 ± 13.9	272.0 ± 11.0	274.6 ± 10.5
III	268.6 ± 11.7	262.7 ± 15.1	262.0 ± 13.9	264.0 ± 12.9	260.5 ± 12.8	267.7 ± 12.8	266.3 ± 13.7
IV	258.0 ± 12.7	253.0 ± 12.2	250.4 ± 19.4	250.2 ± 15.2	252.1 ± 14.4	255.3 ± 15.7	256.9 ± 15.0
V	268.5 ± 7.9	261.9 ± 7.9	278.1 ± 11.4	262.3 ± 7.9	263.6 ± 5.9	273.0 ± 9.2	268.5 ± 5.7

Group I received no treatment; group II was treated with the sea buckthorn seed oil; group III was treated with the DHQ suspension treatment; group IV was treated with a solution of L-lysine monohydrate; group V was treated with a DHQ- L-lysine composition.

At the same time, during the first week of the experiment, a tendency towards a decrease in body weight in rats was observed in all groups, but no significant difference was reached. Body weight returned to the initial value or exceeded it on days 28, 14, and 7 in groups I, II and V, respectively.

The body temperature of animals in all groups on the day of burn formation was 36.9 ± 0.4°C (*p* < 0.05). On day 1, it decreased to 35.4 ± 0.6°C (*p* < 0.05). No other significant difference was observed between the measurements.

On the first day after the burn modelling, the formation of blisters was observed on the damaged skin. The average wound area in all groups was 5.61 ± 0.20 cm^2^ (*p* < 0.05). No significant difference in area was detected between the groups (Table 2).

**TABLE 2:** Absolute wound surface area.

Group	Average area ± half-width of the confidence interval, cm^2^					
	Day 1	Day 7	Day 14	Day 21	Day 28	Day 35
I	5.52 ± 0.54	5.14 ± 0.41	4.51 ± 0.43	2.41 ± 0.46	1.21 ± 0.50	0.59 ± 0.49
II	5.54 ± 0.45	5.60 ± 0.56	4.47 ± 0.75	2.01 ± 0.52	0.99 ± 0.32	0.46 ± 0.25
III	5.75 ± 0.57	5.44 ± 0.34	4.45 ± 0.70	2.68 ± 0.69	0.92 ± 0.40	0.28 ± 0.18
IV	5.73 ± 0.57	5.41 ± 0.33	4.68 ± 0.47	2.85 ± 0.59	1.19 ± 0.24	0.33 ± 0.14
V	5.51 ± 0.42	5.34 ± 0.38	3.90 ± 0.50	1.67 ± 0.47	0.55 ± 0.26	0.11 ± 0.05^a^

^a^ Shows a significant difference from the positive control group, *p* < 0.05.

Group I received no treatment; group II was treated with the sea buckthorn seed oil; group III was treated with the DHQ suspension treatment; group IV was treated with a solution of L-lysine monohydrate; group V was treated with a DHQ- L-lysine composition.

On day 7, scab formation was observed in all groups. A decrease of 7.0, 5.4, 5.5, and 3.1% in absolute values and 6.0, 4.2, 4.1 and 2.8% in relative values was observed in groups I, III, IV, and V, respectively. However, there was no significant difference between the experimental groups during the first week of treatment.

On day 14, active wound healing was observed in group V, which was ahead of the other groups by 9.6 ± 2.0% (*p* < 0.05) in absolute values, and by 9.8 ± 2.1% (*p* < 0.05) in relative area. The remaining groups showed similar results without significant differences in terms of area.

At the end of the third week of treatment, active healing of the wound was observed in groups II and V. In the period from day 14 to day 21, the burn area decreased by 55.0 and 57.2% (absolute values) and by 44.6 and 40.4% (relative values), respectively, compared to the area on day 14. This tendency continued until the end of the experiment. In the period from day 21 to day 28, a significant decrease down to 31.7 and 28.9% in relative area was observed in groups III and IV, respectively.

**FIGURE 3:**
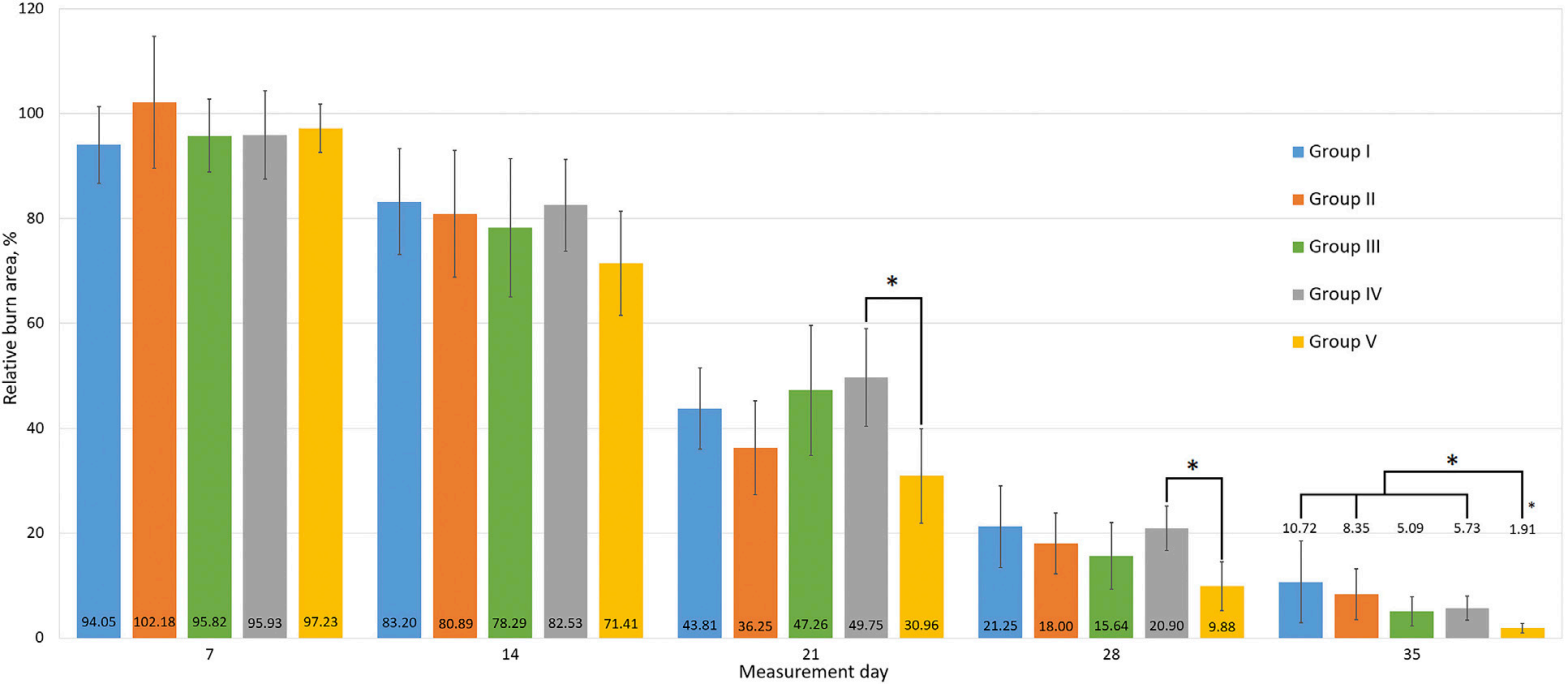
The relative areas of the wound surfaces. All data are presented as mean ± half-width of the confidence interval, with * indicating a significant difference between groups, *p* < 0.05. Group I received no treatment; group II was treated with the sea buckthorn seed oil; group III was treated with the DHQ suspension treatment; group IV was treated with a solution of L-lysine monohydrate; group V was treated with a DHQ- L-lysine composition.

**FIGURE 4:**
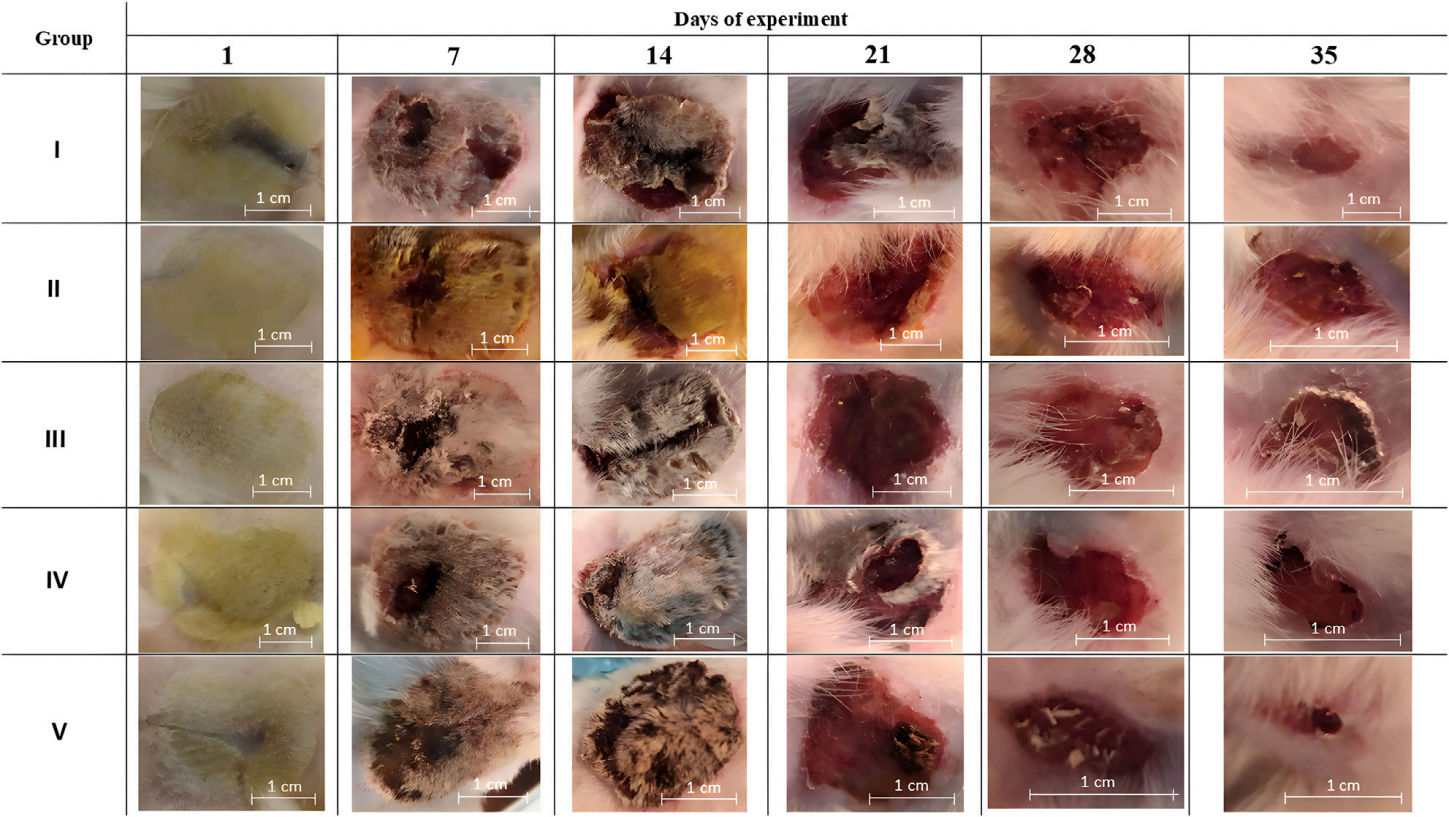
Macrophotographs of the burn surfaces. Group I received no treatment; group II was treated with the sea buckthorn seed oil; group III was treated with the DHQ suspension treatment; group IV was treated with a solution of L-lysine monohydrate; group V was treated with a DHQ- L-lysine composition.

On day 35 a significant difference in the relative burn area was observed in group V (Figure 3). The other treatments did not differ significantly in relative values at the end of the experiment. Although there were tendencies towards more active wound healing in groups III and IV, which have a smaller confidence interval than group V. Macrophotographs for each day of burn surface area measurements are presented in Figure 4.

A two-way ANOVA was performed on the relative burn area values on day 35 to determine the nature of the pharmacological interaction between the components. A significant difference was found in the DHQ treatment group compared to no treatment (*F* = 5.69, *p* = 0.022). Similar results were obtained for the lysine treatment group (*F* = 4.26, *p* = 0.045). For the composition treatment group, only an additive effect of the combined use of the components was observed (*F* = 0.21, *p* = 0.649).

Histochemical analysis was used to study differences in the molecular and cellular mechanisms underlying wound healing in the experimental groups. Its results are presented below.

**FIGURE 5:**
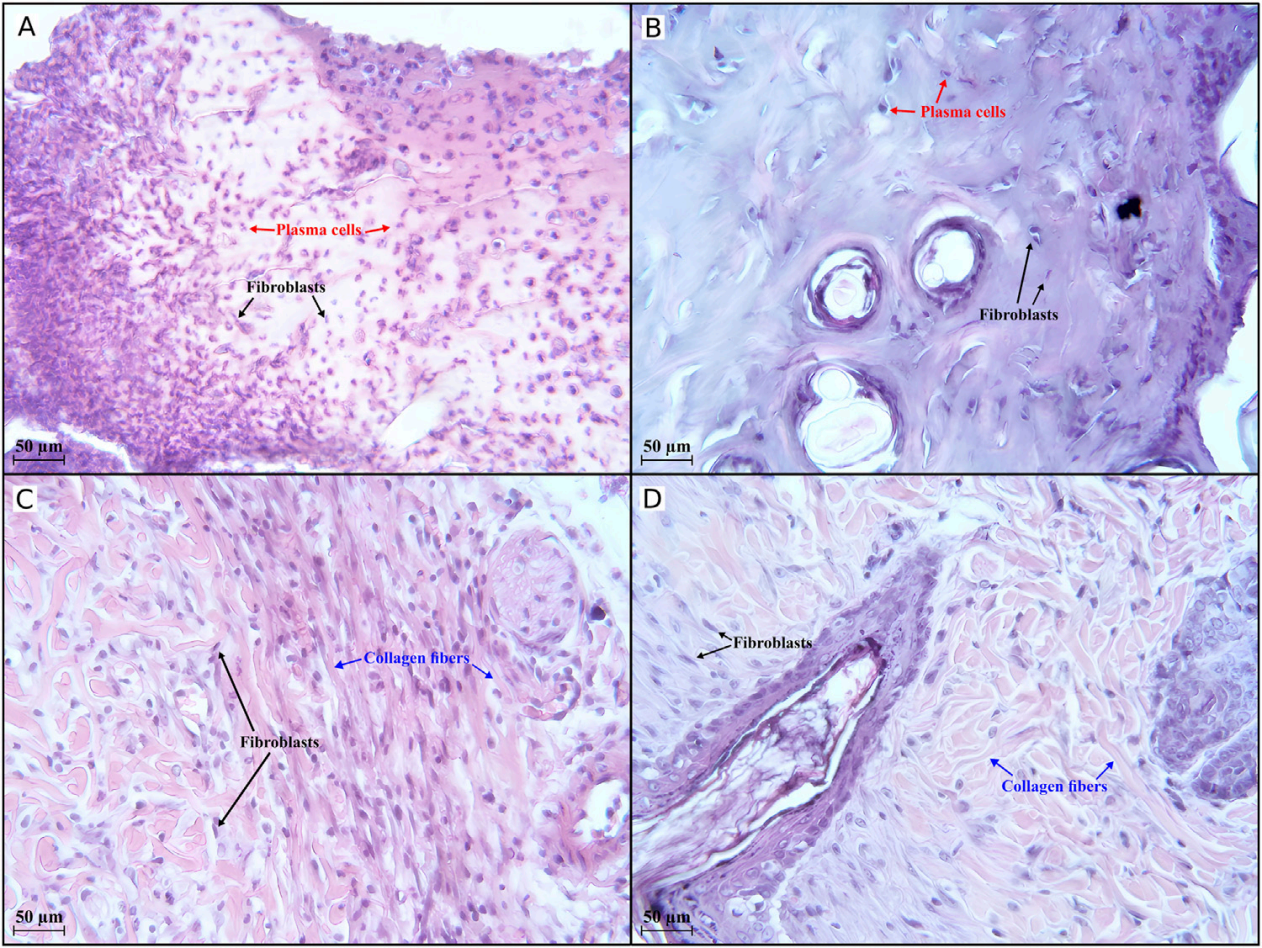
Histological evaluation (×400 magnification) of wound healing in the first half of the experiment. An active inflammatory process is observed in groups I **(A)** and III **(B)** on day 7, with 273 and 59 plasma cells, respectively, in the field of view. In groups IV **(C)** and V **(D)**, an active regenerative process occurs, with 122 and 88 collagen fibres, respectively, visible in the field of view. Group I received no treatment; group II was treated with the sea buckthorn seed oil; group III was treated with the DHQ suspension treatment; group IV was treated with a solution of L-lysine monohydrate; group V was treated with a DHQ- L-lysine composition.

**FIGURE 6:**
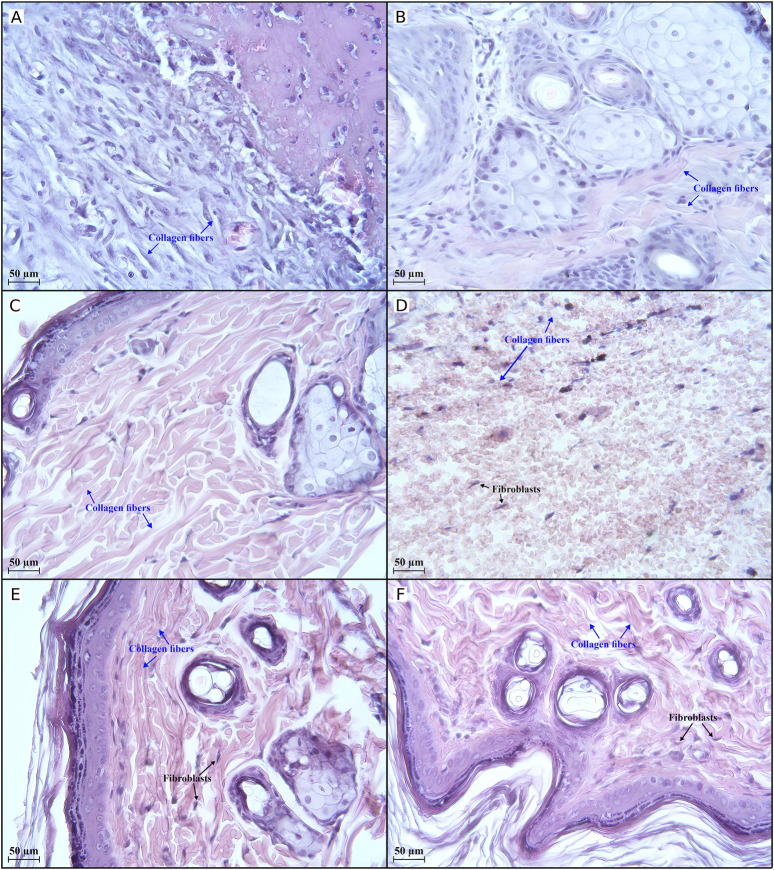
Histological evaluation (×400 magnification) of wound healing in the second half of the experiment. There is an active regenerative process in group II **(A)** on day 21, indicated by 33 collagen fibres in the field of view. Completion of the active regeneration process on day 28 in groups IV **(B)** and V **(C)** was confirmed by 47 and 139 collagen fibres, respectively, in the field of view. The low activity of regenerative processes in group I **(D)** on day 35 is illustrated by 11 collagen fibres in the field of view. Active regenerative processes on day 35 in groups II **(E)** and III **(F)** were confirmed by 119 and 136 collagen fibres, respectively, in the field of view. Group I received no treatment; group II was treated with the sea buckthorn seed oil; group III was treated with the DHQ suspension treatment; group IV was treated with a solution of L-lysine monohydrate; group V was treated with a DHQ- L-lysine composition.

The most representative changes in histology are shown in Figures 5, 6.

Histology in groups I, II, and III on day 7 shows areas of extensive mononuclear infiltration. The dermal area is non-epithelialised and filled with large numbers of fibroblasts and plasma cells (Figures 5A, B).

In group IV, the formation of a poorly differentiated epidermal layer was noted. Fibroblasts and thin collagen fibres were also observed (Figure 5C). A moderate amount of collagen fibres and many fibroblastic cells could be seen in group V. It was also possible to recognise the layers of the epidermis (Figure 5D).

Figure 6A shows an improvement that was observed in group II on day 21: the skin surfaces began to epithelialise, and the border between the dermis and epidermis was relatively smooth. Differentiated layers, fibrillar structures, and venous congestion were observed.

After 1 month of treatment, an increase in the number of collagen fibres, cells around the vessels, and venous plethora was observed in group IV. The process of keloid scar formation was underway (Figure 6B). As shown in Figure 6C, the epidermal and dermal layers in group V were found to be clearly differentiated. A large number of collagen fibres was clearly visible, and an active process of keloid scar formation was noted.

On the last day of the experiment, a weak formation of epidermal tissue was noted in group I. The dermal area was filled with a small number of fibroblasts. Lymphocyte infiltration, haemorrhages, and a small amount of collagen fibres were also observed (Figure 6D). In group II, the number of collagen fibres increased, the number of fibroblastic cells decreased, and a scar began to form (Figure 6E). A similar picture was observed in group III (Figure 6F), and the same histology was observed in group IV and group V on day 28.

## Discussion

The design of this study was aimed at assessing the wound healing activity of the DHQ-L-lysine composition in comparison with the individual components, a herbal medicine approved for the treatment of burns, and a placebo. It is important to note that assessing the pharmacological activity at the pharmaceutical development stage reduces development costs. It also ensures successful translation into clinical practice and accelerates the entry of new drugs into the market [41]. During the experiment, not only absolute values of burn areas were assessed, but also relative values. This allows for a better understanding of the dynamics of wound healing in each group. In addition, the heterogeneity in the initial areas of the wound was insignificant.

As a result of the burn modelling, the experimental animals developed injuries with a clinical picture corresponding to a third-degree burn [42]. This type of thermal injury is one of the most commonly used to assess the wound-healing effects of various drugs [43].

Based on the results of the general condition analysis, it can be assumed that the experimental groups formed as a result of randomisation are characterised by high homogeneity. This, along with the similarity of the morphological structure of the skin of the experimental animals to human skin [44], increases the relevance of the current results. The fluctuations in weight might be caused by discomfort due to the modelled thermal injury to the skin. Similar changes in burn models have been observed by Duan et al. [45] and Lakshmi et al. [46]. The sharp decrease in body temperature in experimental animals after the formation of a burn can be explained by partial removal of hair and impaired thermoregulation [47].

The wound-healing process in group I was the most difficult. Until the last day of the experiment, some animals retained the remnants of a scab. This is also indicated by the slowest decrease in burn surface area of all study groups. On day 7, histological analysis showed the deep appearance of pathological processes caused by the burn, in particular inflammation. Signs of the proliferation stage were observed on day 35, which was the latest of all the experimental groups. Similar results have been observed in other studies [48], where a negative control group was treated with normal saline. However, there are data [49] showing that such treatment is not significantly different from no treatment.

In group II, the histology did not differ significantly from placebo during the first half of the experiment. There were no positive wound healing dynamics during the first week of the experiment, which can be explained by the formation of an oil film on the injured skin surface. This reduces gas exchange between the wound and the environment and makes it difficult to release inflammatory products. As indicated by histochemical analysis, the proliferation stage occurred only on day 21. Sea buckthorn seed oil has previously been studied for its wound-healing effect after thermal burns in the Sprague-Dawley rat model [50], where a significant effect was demonstrated compared to silver sulfadiazine. The authors suggested that this effect was associated with the presence of omega-3 and omega-6 fatty acids, tocopherol, and carotenoids in the oil. In addition, the Merino sheep model [51] showed accelerated wound healing under the influence of sea buckthorn oil. The study supports evidence from previous observations of the beneficial effects of the topical application of sea buckthorn oil in a clinically relevant large animal model.

In group III, wound healing was more active than in the negative control group. At the end of the experiment, almost complete hair regrowth on the damaged dorsal surface was observed in some animals. Such observations are in good agreement with literature sources. There are data [52] on the study of the wound-healing activity of flavonoids in conditions of wound infection with *Staphylococcus aureus*, *Pseudomonas aeruginosa*, and *Candida albicans*. Significant activity of DHQ has been shown in the treatment of such skin lesions. This suggests that the antimicrobial effect of flavonoids may further promote wound healing after various injuries. This property has been further demonstrated in a liposomal dosage form [53], which has strong antioxidant activity and sustained release. A similar antimicrobial effect has not been demonstrated for other flavonoids [54]. Additionally, during DHQ treatment, the onset of proliferation was observed on day 21. In group I, this occurred earlier than in the negative control group, suggesting that this flavonoid is involved in tissue regeneration processes. The results are consistent with histopathological and immunohistochemical data from other scientific groups, which have shown active skin regeneration through regulation of the expression of the CD68, CD31, and VEGF genes [55].

Visually, the wound-healing process in group IV was similar to that in group III. However, according to histological analysis, while the majority of the experimental animals were still in the exudation phase at the end of the first week, the beginning of proliferation was observed in group IV. A possible reason for such an early regeneration process could be the presence of lysine in the treatment course [56, 57].

The most interesting results were obtained in group V, where the wound-healing process was the most active. At the end of the experiment, almost complete regrowth of the damaged surface with new hair was observed in this group. The significant difference in the relative burn area at day 35 clearly indicates the high biological activity of the composition compared to other treatments, that were studied. Moreover, signs of proliferation were observed as early as day 7, as in group IV. On day 28, a clear separation of the epidermal and dermal layers was noted, suggesting the high potential of the composition for tissue regeneration. This also indicates the presence of an additive effect in the wound healing effect due to the combined presence of the flavonoid and the amino acid. Previous studies on the anti-burn activity of DHQ have shown that the rate of wound healing correlates with an improvement in the biological availability of pseudo-polymorphic modifications [9]. However, this did not solve the problem of the low solubility of the compound in water at room temperature, in contrast to the analysed composition.

Interesting results were obtained by semi-quantitative evaluation of histological samples during the assessment of inflammatory status and wound healing stages.

A high number of plasma cells (*n* = 273) was observed in group I on day 7. This suggests an intense inflammatory response at an early stage, indicating an active immune response.

Fifty-nine plasma cells were observed in group II on day 7. Compared to group I, this indicates a less intense inflammatory response during the first week of the experiment. A smoother progression into the proliferative phase can therefore be assumed.

On the contrary, no plasma cells were found in groups IV and V on day 7, indicating the end of the inflammatory process and ongoing tissue regeneration. The number of fibroblasts was 291 in group IV, while it was 115 in group V. Additionally, a high number of collagen fibres was observed in these groups in the early stages of wound healing: 122 and 88 in group IV and group V, respectively. These data indicate the beginning of the proliferative phase and suggest an initially strong regenerative response.

On day 35, compared to day 7, the number of fibroblasts in groups I and II changed from 185 to 44 and from 48 to 36, respectively. The increase in collagen fibres in group II from 33 (day 21) to 119 (day 35) suggests that the tissue is undergoing active remodelling and strengthening, which is a sign of the maturation phase of wound healing. The number of collagen fibres in group I reached only 11 on day 35, which together with the decrease in fibroblasts suggests poor progression into the proliferative and remodelling phases and implies weak tissue regeneration.

On day 35, group III showed a robust presence of fibroblasts (*n* = 64) and collagen fibres (*n* = 136), indicating strong regeneration and effective tissue remodelling. The data suggest that the group had reached the maturation phase successfully.

The results obtained on day 28 in groups IV and V suggest an initially strong regenerative response. However, the decrease in collagen fibres in group IV down to 47 pieces may indicate insufficient maturation of the tissue. At the same time, there was an increase in collagen fibres (from 88 to 139) observed in group V on day 28. This observation indicates that the tissue has progressed into the remodelling phase, where collagen deposition is critical for strengthening and maturing the newly formed tissue. The higher collagen count indicates ongoing tissue repair and successful regeneration [58, 59].

However, as the counting of plasma cells, fibroblasts, and collagen fibres was carried out by a single observer, the results may contain an element of subjectivity. Nevertheless, such approaches are commonly used to determine the stages of the regeneration process. Also, one of the limitations of the experiment was the lack of scoring of the inflammatory effect during the formation of burns. The employing of more comprehensive histopathological techniques, such as specific staining and graded tissue examination, may be beneficial and increase data robustness. Additionally, the inclusion of protein expression analyses like Western blotting could provide objective measurements of wound closure, inflammatory markers, and protein levels associated with wound repair. The lack of protein expression or molecular marker analysis limited our understanding of the exact mechanisms driving the faster wound-healing rate in the DHQ-L-lysine group. When conducting a preclinical study, after obtaining a dosage form, it is of interest to conduct such an assessment.

Moreover, the current experimental model has some limitations associated with physiological and morphological differences between experimental animals and humans. First, unlike humans, rats are characterised by high skin elasticity and weak connection to the underlying tissues. This may affect the wound-healing process and contribute to wound reduction rather than epithelisation [60–62]. In addition, rats produce the enzyme l-gluconolactone [63], which is absent in humans and promotes the synthesis of vitamin C, which is essential for collagen formation. Despite these characteristics, the rat model remains one of the most important ones for the investigation of drugs for the treatment of burns [43].

Despite these limitations, the study demonstrated the high potential of the resulting composition in burn therapy, not only compared with placebo, but also with herbal medicines approved for use in this nosology. In turn, the proposed DHQ-L-lysine composition can be used to develop drugs in the form of a spray, which is practical for outpatient care. Nevertheless, other delivery forms are possible. For example, composite films based on polymer with polyphenols have been actively researched in recent years [53, 64–66]. Due to prolonged wound contact, potential therapeutic effects are amplified. The development of several dosage forms for DHQ will allow healthcare professionals the opportunity to choose the optimal medication for different cases, improving the management of patients with burns and other types of wounds. However, it is important to note that due to the presence of 2 centres of chirality, the DHQ molecule can exist in the form of 4 enantiomers, the pharmacological effects of which may differ [67]. For this reason, when developing drugs based on this active pharmaceutical ingredient, it is necessary to take into account its stereoisomeric composition.

## Conclusion

This research aimed to investigate the potential of a DHQ-L-lysine composition to promote wound healing in a rat burn model. A notable observation from this study is that the topical application of the composition solution appeared to be associated with a higher rate of skin regeneration following third-degree burns. Based on a semi-quantitative analysis of histological samples, the treatment with the DHQ-L-lysine composition appeared to show a trend towards the intensification of reparative processes. Another important finding suggests that this flavonoid and amino acid may exert an additive effect on biological activity.

This project contributes to the growing body of research exploring the potential of natural small molecules in burn therapy. To the best of our knowledge, this represents one of the first efforts to thoroughly evaluate the *in vivo* biological activity of water-soluble DHQ. A logical next step would be to explore the development of a suitable dosage form for the DHQ- L-lysine composition. This area appears to be a promising direction for future investigation.

## Data Availability

The raw data supporting the conclusions of this article will be made available by the authors, without undue reservation.
